# Mediterranean Diet and Sarcopenia Features in Apparently Healthy Adults over 65 Years: A Systematic Review

**DOI:** 10.3390/nu15051104

**Published:** 2023-02-22

**Authors:** Sousana K. Papadopoulou, Paraskevi Detopoulou, Gavriela Voulgaridou, Despoina Tsoumana, Maria Spanoudaki, Faviola Sadikou, Vasiliki G. Papadopoulou, Christiana Zidrou, Ioanna P. Chatziprodromidou, Constantinos Giaginis, Pantelis Nikolaidis

**Affiliations:** 1 Department of Nutritional Sciences and Dietetics, International Hellenic University, 57400 Thessaloniki, Greece; 2 Department of Clinical Nutrition, General Hospital Korgialenio Benakio, 11526 Athens, Greece; 3 Clinical Dietetics & Nutrition Department of 424 General Military Hospital, New Efkarpia Ring Road, 56429 Thessaloniki, Greece; 4 2nd Orthopaedic Department, G. Papageorgiou General Hospital, 54453 Thessaloniki, Greece; 5 Department of Public Health, Medical School, University of Patras, 26504 Patras, Greece; 6 Department of Food Science and Nutrition, School of Environment, University of Aegean, 81400 Myrina, Greece; 7 School of Health and Caring Sciences, University of West Attica, 12243 Athens, Greece

**Keywords:** Mediterranean diet, sarcopenia, muscle strength

## Abstract

Low muscle mass combined with changes in physical function and muscle quality is defined as sarcopenia. In people > 60 years, sarcopenia reaches 10% and tends to increase with age. Individual nutrients, such as protein, may have a protective role against sarcopenia, but recent evidence suggests that protein alone has been ineffective in increasing muscle strength. Dietary patterns, instead, with a high “anti-inflammatory” potential, such as the Mediterranean dietary pattern, have been considered as an emerging dietary remedy against sarcopenia. The aim of this systematic review was to summarize the evidence of the role of Mediterranean diet in sarcopenia prevention and/or improvement, including recent data, in healthy elders. We searched published studies about sarcopenia and the Mediterranean diet until December 2022 in Pubmed, Cochrane, Scopus search engine and grey literature. In total, ten articles were identified as relevant: four cross-sectional studies and six prospective. No clinical trial was identified. Only three studies assessed sarcopenia presence and four measured muscle mass, which is an essential criterion in sarcopenia diagnosis. Mediterranean diet adherence had, in general, a positive role in muscle mass and muscle function, while the results were less clear with regard to muscle strength. Additionally, there was no evidence of a positive effect of the Mediterranean diet on sarcopenia. There is a need for conduction of clinical trials in order to reach cause–effects conclusions regarding the importance of the Mediterranean diet in sarcopenia prevention and management in Mediterranean and non-Mediterranean populations.

## 1. Introduction

Low muscle mass combined with changes in physical function and muscle quality is defined as sarcopenia and is caused by age, chronic disease, physical inactivity or reduced mobility, and malnutrition [1,2]. This condition increases the risk of falls, disability, low quality of life and death [1]. Although the prevalence of sarcopenia depends on the criteria or the cut-off points used [3], a meta-analysis of our group reported that the prevalence of sarcopenia in nursing homes was 31% (women) and 51% (men), in hospitalized patients 23% (men) and 24% (women) and in community 9% (women) and 11% (men) [4]. In people > 60 y, sarcopenia reaches 10% prevalence and tends to increase with age [5].

Food quantity and quality, as well as consumed energy, may be considered as preventive factors of sarcopenia. A repeatedly reduced energy intake may lead to muscle atrophy, muscle fatigue, weakness and frailty. During periods of poor feeding, adipose tissue and muscle mass are reduced [1]. The latter is particularly difficult to regain, especially in older people [6]. Instead, adequate protein consumption can improve muscle strength and mass in healthy older adults [7], as well as in aging, inflammation and disease states [8]. In this context, a European Society for Clinical Nutrition and Metabolism (ESPEN) expert group recommends an intake of 1.0–1.2 g protein/kg body weight/day for healthy older adults and 1.2–1.5 g protein/kg for malnourished older adults [9]. However, a protein stand-alone intervention was ineffective in increasing muscle strength in older adults [10]. 

Muscle loss may worsen in chronic inflammation [11]. An “anti-inflammatory diet” may be thus considered beneficial for subjects with sarcopenia, as there are currently no approved medical treatments for this condition [1]. Many researchers focus on the intake of antioxidants from the diet and their effects on muscle mass and/or function, which are affected by the aging process. For example, low selenium, vitamin C and E levels may be connected to reduced muscle mass in older subjects [12]. In contrast, increased circulating carotenoid concentration is associated with a lower risk of severe kinetic disability among the elderly [12]. *N*-3 fatty acids also have anti-inflammatory properties [13], while saturated fatty acids may increase inflammation [14]. Since reduced circulating *n*-3 levels has been connected to sarcopenia presence, a good fatty acid profile could enhance human muscle homeostasis [12]. In parallel, antioxidant micronutrients together with *n*-3 may protect against other diseases in the elderly [15,16], which may in turn affect inflammatory burden as well as the risk of malnutrition and sarcopenia [17].

However, individual nutrients or foods may not be as important as a comprehensive dietary pattern and/or meal pattern, since nutrients are consumed in combination, interact with each other and influence health status [18]. In this context, it is vital to identify which nutritional regimen will be most helpful in sarcopenia because people eat foods, not nutrients [19]. The Mediterranean diet consists of antioxidants, anti-inflammatory micronutrients and *n*-3 fatty acids and is characterized by a high intake of monounsaturated fat and fiber [20,21]. Moreover, it includes vegetables including green leafy vegetables, fruits, fish, healthy fats, and mainly olive oil, legumes, whole grains, nuts and seeds, moderate intake of dairy products and wine consumption as well as low consumption of processed foods, confectionery and red meat [22,23].

High adherence to the Mediterranean diet is associated with a lower incidence of chronic diseases and lower physical impairment characteristics of aging [24] and frailty [25]. The Mediterranean diet also exerts protective effects against platelet aggregation [26,27], cardiometabolic risk [28], diabetes [29], mental disorders, including cognitive decline [30,31] and cancer [32]. Its beneficial effects seem to be exerted in both populations of Mediterranean and non-Mediterranean areas [33]. According to Granic et al., the adherence to a Mediterranean diet has a myo-protective effect [19]. In fact, in weight loss programs based on the Mediterranean diet, decreased loss of lean tissue is documented [34,35]. Indeed, the study of Barrea et al. showed that adherence to the Mediterranean diet was positively related to muscle function, as assessed by handgrip strength in active elderly women [36]. 

To our knowledge, several systematic [37,38,39,40,41,42,43] and narrative reviews [19,24,44,45] have focused on dietary patterns and sarcopenia. Some of them have grouped all healthy dietary patterns together [37,39] or were not conducted in older adults [37,40]. Other reviews were focused on the role of diet and/or dietary patterns in sarcopenia in pathological states, such as cancer [46,47]. The latest systematic review and meta-analysis examined healthy dietary patterns, in general, in relation to sarcopenia [39] or had a special focus on developing countries [43]. None of the latest works, however, assessed the Mediterranean diet as a distinct ontology [39,43], except the systematic review of Jang et al., in which only prospective cohort studies were included [42]. Last but not least, several recent studies have been published [48,49], which were not considered in the previous reviews, the majority of which (8 out of 11) were published up to 2020 [19,24,37,38,40,41,44,45].

The aim of this systematic review was to summarize the evidence of the role of the Mediterranean diet in sarcopenia prevention and/or improvement, including recent data, in healthy elders. To the best of our knowledge, this is the first review of the published literature synthesizing data on the Mediterranean diet and sarcopenia in healthy aging individuals.

## 2. Materials and Methods

This systematic review was conducted according to the Preferred Reporting Items for Systematic Review and Meta-Analyses (PRISMA) guidelines [50] and the study protocol was registered in the PROSPERO (acknowledgement of receipt No: 396143; registration number: pending). We searched published studies about sarcopenia and the Mediterranean diet from 2000 until December 2022 in Pubmed, Cochrane, Scopus search engine and grey literature. We also searched the references of relative reviews for additional articles. The search strategy used combined results of the following key words: (1) Mediterranean diet (“mediterranean diet” OR “med diet”) and (2) Sarcopenia (sarcopenia OR “muscle mass” OR “muscle strength” OR “physical function”). The results of each strategy of a term were combined with the Boolean Operator “AND”. Τhe final search strategy was formulated as follows. Table 1 presents the search strategy in detail. No language restriction on the retrieved articles was applied.

The research question was formulated as follows: population (P), intervention (I), outcome (O). Table 2 describes in detail the research question.

### 2.1. Inclusion and Exclusion Criteria

Inclusion criteria were (1) randomized control trials or observational studies, (2) healthy elders aged ≥ 65 years old, (3) Mediterranean diet in observational studies should be investigated through validated methods. 

Exclusion criteria were (1) studies performed on animals, (2) Letter to the Editor, case series, case reports, (3) participants with previously diagnosed disease.

### 2.2. Study Selection

Two independent investigators (P.D. and D.T.) screened all the retrieved articles by title and abstract at the initial screening stage in order to exclude irrelevant articles. The two investigators independently read the full texts of the articles that were not excluded from the initial stage and they selected studies that met the inclusion criteria for the systematic review. Any disagreement regarding the selection of the articles was resolved by consensus between both two investigators together with a third co-author (G.V.). 

### 2.3. Quality Assessment

The quality assessment of the studies was conducted by two researchers independently (P.D. and D.T.) using the New Castle Ottawa scale (NOS) [51,52] for prospective, cohort and case–control studies, while the critical appraisal tool to assess the cross-sectional studies was also used (AXIS) [53]. A third researcher (G.V.) intervened when no agreement was reached between the researchers.

### 2.4. Data Extraction

Two independent researchers (P.D. and D.T.) performed data extraction in predefined excel spreadsheets. Any disagreements were discussed with a third researcher (G.V.). The following items were extracted from each study: study details (first author, year of publication, design), sample details (number of participants, age, sex, ethnicity), assessment of sarcopenia and surrogate measures, prevalence of sarcopenia, follow-up, Mediterranean tools assessment, results (outcomes about muscle mass, muscle strength, physical function and other secondary outcomes related to correlations of other dietary components). 

### 2.5. Data Synthesis

No maximum or minimum sample size requirement of included studies was applied. 

It was not possible to perform a meta-analysis due to the variability in the tools used for body assessment and/or muscle function. Thus, a narrative synthesis was performed. The variables assessed were sarcopenia or the surrogate measures of sarcopenia, such as muscle mass, muscle strength and physical function. The intervention variable was the Mediterranean diet.

## 3. Results

**Figure 1:**
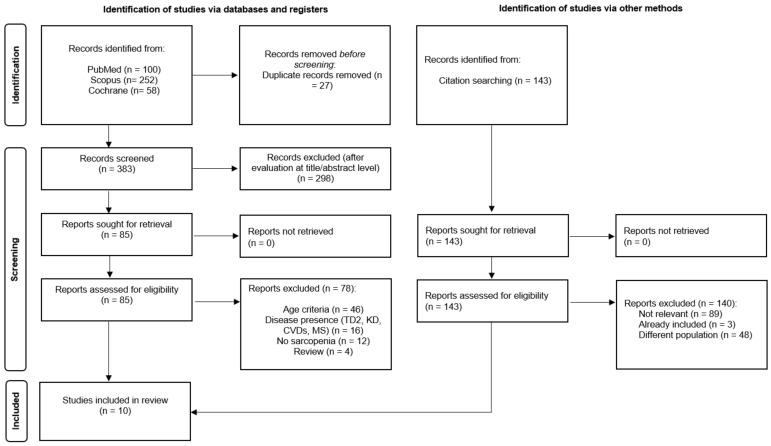
PRISMA [50] flowchart of studies’ selection process.

Out of a total of 553 search results, 10 fulfilled the inclusion criteria and were included in the present systematic review (Figure 1). Four cross-sectional studies [48,54,55,56] and 6 prospective studies [49,57,58,59,60,61] were included in this review. Table 3 details the characteristics of the included studies.

A total of 10 studies provided data about the relation of Mediterranean diet with sarcopenia in elderly individuals (*n* = 17,663) [48,49,54,55,56,57,58,59,60]. The majority of studies included both males and females [48,54,55,56,57,58,59], except from one study including exclusively women [60] and one including only men [49]. The studies took place in the USA [58], Korea [54], Australia [49], Hong Kong [61] and some European countries [49,55,56,57,59,60]. No clinical trial was identified. Participants’ mean age ranged from 73 to 86 years, but some studies have not reported mean values [54,60,61,62]. Dietary intake was assessed with different types of FFQ [48,55,57,58,59,61,62], 24 h recall [54], 3-day record [60] and validated diet history [49]. The adherence to the Mediterranean diet was assessed with aMED [54], MEDAS [48], PREDIMED questionnaire [56], MSDPS [55], MEDI-LITE score [49], MED score [60,61] and DQI-I [57,58,59,60]. Several measures related to sarcopenia were assessed, such as handgrip strength [54,55,56,59] or/and sitting time [56], SPPB [55,57], or even appendicular lean mass (ALM) [48]. Sarcopenia was assessed with the criteria of EWGSOP2 [49,60], SARC-F/SARC- [48] or those of the Asian Working Group [61]. It is noted that the study of Cervo et al. is presented both regarding the relation of the Mediterranean diet with muscle mass and function (sarcopenia features) [49] and the relation of the Mediterranean diet with sarcopenia, since valid criteria were used for the assessment of the latter [49].

### 3.1. Mediterranean Diet and Muscle Mass

Only two studies of prospective design investigated the relation of Mediterranean diet adherence with muscle mass [49,60]. In the study of Cervo et al., a higher MedScore score was associated with higher appendicular lean mass [49] and in the study of Isanejad et al., women with higher adherence to the Mediterranean diet had lower skeletal muscle index and lean mass loss [60].

### 3.2. Mediterranean Diet and Muscle Strength

Four studies assessed muscle strength, three cross-sectional [54,55,62] and one prospective [59] study. The results were mixed, since some studies showed a positive relation of the Mediterranean diet with handgrip strength [54,62], while others documented no significant relation [55,59]. 

### 3.3. Mediterranean Diet and Muscle Function

In total, six studies assessed muscle function in relation to Mediterranean diet adherence, with cross-sectional [62] and prospective design [49,57,58,59,60]. Several tests were used, such as sitting time, Short Physical Performance Battery, etc. All but one documented a positive effect of the Mediterranean diet regarding muscle function, 20 m walking test [57] and 15 ft walking test [59]. 

### 3.4. Mediterranean Diet and Sarcopenia

In the study of Chan, no association between diet and sarcopenia was observed [61]. Higher adherence to the Mediterranean diet was related to faster walking speed 10 m, greater LBMQ and better performance regarding squat test completion [60]. The study of Borges et al. assessed sarcopenia and Mediterranean diet adherence but no association was found between adherence to the Mediterranean diet between sarcopenic and non-sarcopenic individuals [48].

### 3.5. Quality Assessment of the Included Studies

The quality assessment of cohort studies according to the New Castle Ottawa scale (NOS) is presented in Supplementary Table S1. The overall quality score was satisfactory with three studies having six stars and seven studies having seven stars in the NOS. All the included studies have an appropriate study design. Only one study justified the sample size. The quality assessment of the four cross-sectional studies based on the AXIS tool is presented in Supplementary Table S2.

**Table 3:** Studies investigating the relation of Mediterranean diet and sarcopenia features in apparently healthy older adults.

Study	Total n (Females %, Males %)	Country	Age (Years) (Mean ± SD)	Study Design	Assessment of Sarcopenia or Surrogate Measures	Method of Dietary Assessment	Med Diet Score	Follow-Up	Muscle Mass	Muscle Strength	Muscle Function	Other
[54] Kim et al., 2019	3675 (F: 53.5%, M: 46.5%)	Korea	≥65	Cross-sectional	Handgrip strength	Single 24 h recall	aMED	-		Higher diet scores were related to 32–53% lower odds of low handgrip strength		
[62] Mendes et al., 2020	1491 older adults (F: 58%, M: 42%)	Portugal	≥65	Cross-sectional	Handgrip strength Sitting time	14-item FFQ	PREDIMED questionnaire	-		Lower MedScore was associated with low handgrip strength [OR: 1.50; 95% CI: 1.09–2.05].	Lower MedScore was associated with longer sitting time [OR: 1.43; 95% CI: 1.04–1.96].	
[48] Borges et al., 2022	90 (F: 89%, M: 11%)	Spain	≥65 Mean age 83.4 ± 7.2	Cross-sectional	EWGSOP2 SARC-F and SARC-CalF	14-item FFQ	MEDAS score	-	Sarcopenia was related to BMI [OR: 0.79; 95% CI: 0.68–0.91, *p* < 0.05] and calf circumference [OR:0.64; 95% CI: 0.51–0.81, *p* < 0.01]. Calf circumference predicted sarcopenia presence. No relation with Mediterranean diet was documented. Sarcopenia was present in 30% of patients with hip fracture.			
[55] Fougère et al., 2015	304 (F: 59.5%, M: 40.4%)	Italy	>77 Mean 86.3 ± 6.8	Cross-sectional	Short Physical Performance Battery (SPPB) Handgrip strength	13-item FFQ	Mediterranean diet score (MSDPS)	7 and 10 years		No correlation reported between diet and handgrip strength.	Higher MedScore was associated with better performance at lower limbs (SPPB > 7).	
[49] Cervo et al., 2021	794 (F: 0%, M: 100%)	Australia	81.1 ± 4.5	Prospective	Appendicular lean mass (ALM) and bone mineral density (BMD) were measured with DXA. Gait speed was assessed by 6 min walking test. Handgrip strength was measured with a dynamometer.	Validated diet history	MEDI-LITE score	5 years 616 participants, 3 years later	Higher MedScore score related to higher appendicular lean mass adjusted for body mass index (ALMBMI) (β: 0.004 kg/kg/m^2^; 95% CI: 0.000, 0.008).		MedScore was not associated with muscle function.	Higher MUFA and MUFA/SFA were associated with 24%, and 28% lower risk of falls in older men, correspondingly. Higher MedScore was associated with lower interleukin-7 (β: −0.017 pg/mL; 95% CI: −0.031, −0.003), and incident falls rates (IRR: 0.94; 95% CI: 0.89, 0.99). MedScore was not associated with bone mineral density.
[60] Isanejad et al., 2018	554 (F: 100%, M: 0%)	Finland	65–72	Prospective	EWGSOP2	3-day record	MED score	3 years	Women in the higher quartile MED scores lost less relative skeletal muscle index and total body lean mass (*p* trend ≤ 0.034).		Higher adherence to Mediterranean diet was related to faster walking speed 10 m, greater LBMQ and better performance in squat tests.	
[61] Chan et al., 2016	6905 (F: 42.6%, M: 57.3%)	Hong Kong	≥65	Prospective	Asian Working Group for Sarcopenia	Valid semi quantitative FFQ	Diet Quality Index-International (DQI-I) and the Mediterranean Diet Score (MDS)	4 years	No association between dietary patterns and prevalent sarcopenia in women No association between dietary patterns and incident sarcopenia.			
[57] Milaneschi et al., 2011	935 (F: 55.6%, M: 44.4%)	Italy	74.1 ± 6.8	Prospective	Short Physical Performance Battery (SPPB)	Valid semi quantitative FFQ	MDS	3, 6, 9 years			Participants with higher adherence exhibited less decline in SPPB score, which was of 0.9 points higher at the 3-year follow-up, 1.1 points higher at the 6-year follow-up and 0.9 points higher at the 9-year follow-up (all *p* < 0.05).	
[58] Shahar et al., 2012	2225 F: 54.1% M: 45.9%	USA	74.5 ± 2.8	Prospective	20 m walking test	Valid semi quantitative FFQ	MDS	8 years			Higher MedDiet adherence was an independent predictor of less decline in usual 20 m walking speed.	
[59] Talegawkar et al., 2012	690 F: 51.7% M: 48.3%	Italy	73 ± 6.24	Prospective	Hand-grip strength 15 ft (4.57 m) walking test	Valid semi quantitative FFQ	MDS	6 years		Ns association with muscle strength	A higher adherence to a Mediterranean-style diet at baseline was associated with low walking speed	

EWGSOP2: European Working Group on Sarcopenia in Older People; CI: confidence interval; F: females; FFQ: Food Frequency Questionnaire; iADL: Instrumental Activities of Daily Living Scale; M: males; OR: odds ratio; U: unspecified; ns: non-significant.

## 4. Discussion

The present systematic review summarized the evidence of the role of the Mediterranean diet in sarcopenia prevention and/or improvement, including recent data, in healthy elders. In total, 10 studies were included, but none were a clinical trial. Only three studies assessed the presence of sarcopenia [48,49,61] and these used different criteria, i.e., EWGSOP2, SARC-F and SARC-CalF [48,49] and the criteria of the Asian Working Group for Sarcopenia [61]. The remaining studies assessed only muscle strength [54], function [57] or combinations thereof with or without muscle mass [48,49,55,56,58,59,60]. Mediterranean diet adherence had, in general, a positive role in muscle mass and muscle function, while the results were less clear with regard to muscle strength. Additionally, there was no evidence of a positive effect of the Mediterranean diet on sarcopenia.

The definition of sarcopenia is a debated issue and several criteria have been developed [1,2]. The diagnosis of sarcopenia may thus differ according to the used method [1]. Low muscle mass is a diagnostic criterion of sarcopenia [1], also adopted by ESPEN [63], but in the present review only few studies have actually measured muscle mass [48,49,60,61]. It is possible that in the epidemiological context, the cost and availability of muscle mass measurements may constitute a limiting factor [2]. The majority of the included studies have thus assessed “sarcopenia-related” measures, such as muscle function and muscle strength rather than sarcopenia per se.

So far, most studies that have evaluated the role of diet in sarcopenia have focused on individual nutrients [12,47]. However, it is very important to understand that a nutritional regimen in sarcopenia will be most helpful because people eat foods, not nutrients. Evidence points to the protective effects of the Mediterranean diet in the elderly [64]. Several scores of Mediterranean diet adherence have been developed, such as the MedDietScore [65], the Mediterranean Diet score (MDS) [66] and its alternated modified version (aMED) [67], the Mediterranean Diet Adherence Screener (MEDAS) for assessing Mediterranean diet adherence among Spanish men and women of older age [68], the Mediterranean-Style Dietary Pattern Score (MSDPS) [69], the MED and MEDI-LITE score [70,71] and that used in the PREDIMED study [72]. Several culture-specific (the Croatian Mediterranean Diet Serving Score, MDSS [73] or the German version of MEDAS [74]) or age-specific (KIDMED) [75] scores have been launched. In the studies reviewed, several valid scores were used; specifically, these were aMED [54], MEDAS [48], PREDIMED questionnaire [56], MSDPS [55], MEDI-LITE score [49], MED score [60], and MDS [61]. The scores were calculated though a highly heterogenous diet methodology, i.e., single 24 h recall [54], 3-day food record [60], short 13- or 14-item FFQ [48,55,62], valid FFQ [61] or the diet history method [49]. Irrespective of the particular index used to assess Mediterranean diet adherence, in the present review beneficial effects of this dietary scheme were recorded.

The Mediterranean diet includes large quantities of olive oil, fruits and vegetables, legumes, nuts, cereals, and fish, low-to-moderate quantity of dairy and low intake of meat [22,23]. It is high in monounsaturated fat, fiber antioxidants, *n*-3 fatty acids and other anti-inflammatory micronutrients [20,21]. The Mediterranean diet exerts anti-inflammatory and immune-regulating properties and has protective effects against platelet aggregation [26,27], cardiometabolic risk [28], diabetes [29], mental disorders, [30,31] and cancer [32]. Several food groups, which are consumed in the context of the Mediterranean diet, such as fruit and vegetable intake, have been inversely related to sarcopenia or its features [60,61,76,77,78]. Indeed, fruits and vegetables contain carotenoids, antioxidant vitamins and phytochemicals [79,80], and they lower oxidative stress and inflammatory burden, which are implicated in the etiopathology of sarcopenia [17]. In fact, oxidative stress increases proteolysis and decreases muscle anabolic procedures, resulting in a reduction in muscle mass [17]. Additionally, nitrates are found in several vegetables. In addition, sarcopenic subjects tend to have lower intake of meat, fish, eggs and legumes, higher intake of vegetables [80], as well as lower intake of nuts and seeds, meat and dairy (males) [81]. Bioactive compounds from whole grains such as polyphenols, β-sitosterol, alkylresorcinols, β-glucan and others can also support muscle anabolism, as recently reviewed [82]. Fish intake, which is a central component of the Mediterranean diet, can positively affect muscle metabolism, since it contains omega-3 fatty acids, lean proteins, selenium, vitamin D [83] and other constituents, such as polar lipids with anti-inflammatory potential against platelet-activating factor [84]. Extra virgin olive oil consumption can stimulate protein synthesis and may have a role in sarcopenia management [85]. For example, rats treated with an olive plus algae oil mixture containing 75% extra virgin olive oil have higher gastrocnemius weight compared to controls [86], while hydroxy-tyrosol inhibits apoptosis in muscles of mice [87]. On the contrary, alcohol [60] and red meat [88] have been associated with sarcopenia and frailty prevalence, correspondingly, while ultra-processed foods have been connected to adiposity [89] and frailty [88,90]. 

Specific food groups containing dietary fiber [79] and the Mediterranean dietary pattern [91] can alter gut microbiota, which has an interplay with muscle phenotype [92]. The role of the Mediterranean diet in immunity [15] may also protect elderly individuals and reduce the need for antibiotic treatment, which disturbs the gut microbiome and may induce muscle atrophy, according to animal studies [93], and also predisposes to sarcopenia.

However, the Mediterranean diet is a dietary scheme low in animal protein. Indeed, many animal protein foods, and especially red meat, are negatively scored [65,66,68], while fish is positively scored [65,66]. There is a debate in the literature on the role of protein origin and its effects on sarcopenia, since animal and plant proteins have a different digestibility and a potentially different effect on muscle protein synthesis stimulation [94]. However, results from a recent meta-analysis showed that animal and plant proteins were equivalent in their effects on lean mass or muscle strength [95]. In parallel, subjects with low total dietary protein also displayed low appendicular lean mass and quadriceps strength, irrespective of the followed dietary pattern [96].

Moreover, the diet–sarcopenia phenotype relationship may be affected by the inflammatory state of participants. Although healthy subjects were included in the present review, sub-clinical inflammation may still apply in the included subjects aged > 65 y. After menopause or andropause, IL-6 increase is associated with decreased lean body mass and frailty [97] and is related, together with C-reactive protein, to a low appendicular lean mass to BMI ratio [98]. In a study of our group, lipoprotein-associated phospholipase A2, a potential anti-inflammatory enzyme in healthy [99] and diseased states [100], was inversely related with lean mass in apparently healthy volunteers of a wide age range [101]. Interestingly, the diet fatty acid and antioxidant profile is a regulating factor of these inflammatory indices in healthy states [14,102]. 

The Mediterranean diet was found to be related to muscle mass in elder subjects according to the presented data. From the included prospective studies, most studies have not tested this hypothesis [57,59,61], while two studies showed positive associations with appendicular lean mass [49] or lower skeletal muscle index [60] and negative association with lean mass loss [60]. The presented studies do not report findings on weight changes. However, it has been shown that the adoption of the Mediterranean diet is particularly important in subjects with comorbidities who may need to lose weight [103], since fat mass loss can be achieved with maintenance of muscle mass [34].

Other diet correlates may also affect the observed relationships. Indeed, subjects with a healthy diet may also have a healthy lifestyle, better social and physical environment [104], healthier meal patterns [18] and an active way of life [105], which may reduce the age-related decline of muscle mass and function [106] and obesity prevalence [107]. It is also noteworthy that a Mediterranean dietary pattern is positively correlated with other measures of diet quality, such as the novel food compass score which assesses the nutrient content of the diet as well as the presence of processed foods in the diet [108].

The adherence to the Mediterranean diet and Mediterranean way of life (such as social networks, involvement in physical activities and sleep quality) have also been connected to better cognitive function and decreased prevalence of dementia [30,31]. This relation is important in light of recent evidence that subjects with dementia may lose their independence and suffer from frailty and sarcopenia [109]. Interestingly, in patients with Alzheimer’s disease, brain atrophy has been associated with decreases in lean mass [110].

The country of origin may play a role, although more data are needed in this direction. All studies found a significant association of the Mediterranean diet with the assessed sarcopenia-related parameters except one, which was conducted in Hong Kong [62]. In a study conducted in Australia, no relation was documented between the Mediterranean diet and muscle function [49]. However, non-significant results in muscle strength were also reported in two Italian studies [55,59]. 

Several limitations should be considered along with the interpretation of the presented results. Although valid tools were used to assess diet and health outcomes, methods differed substantially and food grouping was not standardized. Dietary assessment errors, such as portion estimation errors, memory errors and over- or underestimation of intake, are also inherent in diet–disease research [111]. Moreover, underreporting is a factor that studies can control for [18], but the issue was not addressed in the included studies. In addition, the potential use of dietary supplements (protein or energy drinks) was not assessed in the reviewed studies. These products may be prescribed in malnourished elderly or subjects at risk of malnutrition and their energy and protein content could affect the investigated outcomes [112]. Moreover, the present systematic review included studies in older adults who were apparently healthy. Thus, it is possible that in cases of existing co-morbidities, the magnitude of the Mediterranean diet’s effects may be different since disease-related inflammation and/or mobility impairment may exacerbate sarcopenia and its related features [11]. 

Regarding the methodological quality of the included studies, it is highlighted that the available data were only of cross-sectional and prospective nature. Cross-sectional data have the inherent limitation that they cannot be used to draw cause–effect conclusions. On top of this, an inverse epidemiology effect cannot be ruled out, since subjects with deteriorating muscle strength may have changed their dietary habits towards a better quality. Moreover, in quality assessment, several biases were present, such as the non-responder bias. With regard to prospective studies, it should be noted that dietary habits may change over time. For example, in a Greek population, less than 20% of the subjects sustained a high adherence to a Mediterranean diet over a decade, and on top this volunteers were mostly young [113]. The problems mentioned above can be omitted only in clinical trials, but so far no clinical trial has been conducted on this issue.

## 5. Future Research Directions

In the future, appropriately designed clinical trials are needed, especially in elderly subjects. More particularly, interventions based on the Mediterranean diet should be considered, with adequate intervention periods. Based on other nutrition-related interventions, this period should be at least 4–12 months in order to achieve measurable outcomes on sarcopenia features [114,115]. Additional studies can be designed to test whether a Mediterranean dietary plan, containing specific protein foods, such as dairy, can combat sarcopenia. In this case, a modified MedDiet Score could be proposed, which can be oriented to sarcopenia prevention/and or management. Last but not least, a Mediterranean diet lifestyle (diet, adequate sleep, physical exercise) can be tested in sarcopenia management.

## 6. Conclusions

In conclusion, Mediterranean diet adherence, in general, had a positive role in muscle mass, physical function and sarcopenia, while the results were less clear with regard to muscle strength. Regarding the effect of the Mediterranean diet on sarcopenia, no positive effect was observed. The presented data are based on cross-sectional and prospective studies and only a few studies assessed sarcopenia presence and/or muscle mass. Thus, there is a need for clinical trials to be conducted in order to reach cause–effect conclusions focusing on the importance of Mediterranean diet adoption in sarcopenia prevention and management in Mediterranean and non-Mediterranean populations.

## Figures and Tables

**Table 1:** Search strategy on Pubmed.

#1 sarcopenia OR “muscle mass” OR “muscle strength” OR “physical function”
#2 “Mediterranean diet” OR “Med Diet”
#3 review
#1 AND #2 NOT #3

#: number.

**Table 2:** Formulation of research question (population, intervention, outcome).

P-opulation
Healthy older adults ≥ 65 years old
I-ntervention
Mediterranean diet, Defined by validated questionnaires (i.e., Med Diet Score, etc.)
O-utcome
Sarcopenia Defined by criteria of sarcopenia (i.e., EWGSOP, etc.) or by surrogate measures of sarcopenia (i.e., low muscle mass, low muscle strength, low physical function)

## Data Availability

Not applicable.

## Supplementary Materials

The following supporting information can be downloaded at https://www.mdpi.com/article/10.3390/nu15051104/s1, Table S1: Quality assessment of cohort studies according to the New Castle Ottawa scale (NOS); Table S2: Quality assessment of cross-sectional studies according to the AXIS tool.
