# Supplementary material for: Mediterranean Diet and Sarcopenia Features in Apparently Healthy Adults over 65 Years: A Systematic Review
Source: Nutrients. 2023 Feb 22;15(5):1104. doi: 10.3390/nu15051104 (PMC10005300; doi:10.3390/nu15051104)
Supplement: Supplementary file 1 [file nutrients-15-01104-s001.zip › nutrients-2206122-supplementary.pdf]

nutrients

MDPI

Supplementary Materials

Mediterranean Diet and Sarcopenia Features in Apparently Healthy Adults over 65 Years: A Systematic Review

Sousana K. Papadopoulou [1,*], Paraskevi Detopoulou [2], Gavriela Voulgaridou [1], Despoina Tsoumana [1], Maria Spanoudaki [1,3], Faviola Sadikou [2], Vasiliki G. Papadopoulou [1], Christiana Zidrou [4], Ioanna P. Chatziprodromidou [5], Constantinos Giaginis [6] and Pantelis Nikolaidis [7,*]

Table S1. Quality assessment of cohort studies according to the New Castle Ottawa scale (NOS).

Author	Milaneschi et al. 2011 [56]	Shahar et al. 2012 [57]	Talegawkar et al. 2012 [58]	Chan et al. 2016 [60]	Isanejad et al. 2017 [59]	Cervo et al. 2021 [49]
**Selection**						
1) Representativeness of the exposed cohort	★	★	★	★	★	★
2) Selection of the non-exposed cohort	★	★	★	★	★	★
3) Ascertainment of exposure†	a	a	a	a	a	a
4) Demonstration that outcome of interest was not present at start of study	b	d	Not clear f	Not clear g	h	i
**Comparability**						
1) Comparability of cohorts on the basis of the design or analysis	★★	★★	★★	★★	★★	★★
**Outcome**						
1) Assessment of outcome ††	★	★	★	★	★	★
2) Was follow-up long enough for outcomes to occur‡	★	★	★	★	★	★
3) Adequacy of follow up of cohorts	c	e	★	★	★	j
Total quality score	6	6	7	7	7	6

†A star was given if the exposure was based on medical record or direct measurement.

†† A star was given if the outcome was an independent blind assessment, medical record or direct measurement.

‡ A 3-year follow-up was considered enough for outcomes to occur.

a Dietary assessment was done with a food frequency questionnaire (self-report).

b The Short Physical Performance Battery test was performed at baseline and follow up. However, there was a group free of baseline mobility disability (SPPB score ≥10) in which a higher adherence to Mediterranean diet at baseline was related to lower probability of mobility disability at follow-up.

c From 937 participants 247 participants died, and 10 were lost to follow-up. Participants who were not followed up, were older, more likely to be disabled, reported more chronic diseases, had poorer cognitive function and lower extremity performance.

d Eligibility criteria included self-reporting of no mobility problems.

e The follow-up included 54% of the initial sample after 8 years. The missing subjects were older.

f Not clear. The authors do not report if they made frailty measurements at baseline.

g Sarcopenia was present in several subjects at baseline. Some analysis were done after exclusion of sarcopenic subjects at baseline.

h Sarcopenia was present in several subjects at baseline.

i Measurements performed at baseline and follow-up. Not clear if subjects were excluded from further analysis.

j 22% of the participants were lost at follow-up.

Table S2. Quality assessment of cross-sectional studies according to the AXIS tool.

	Kim et al. 2019 [53]	Mendes et al. 2019 [61]	Borges et al. 2022 [48]	Fougère et al. 2015 [54]
1. Were the aims/objectives of the study clear?	Yes	Yes	Yes	Yes
2. Was the study design appropriate for the stated aim(s)?	Yes	Yes	Yes	Yes
3. Was the sample size justified?	Yes†	Yes	No	No
4. Was the target/reference population clearly defined? (Is it clear who the research was about?)	Yes	Yes	Yes	Yes
5. Was the sample frame taken from an appropriate population base so that it closely represented the target/reference population under investigation?	Yes	Yes	Not Known	Yes
6. Was the selection process likely to select subjects/participants that were representative of the target/reference population under investigation?	Yes	Yes	Not Known	Yes
7. Were measures undertaken to address and categorize non-responders?	Yes	No	No	No
8. Were the risk factor and outcome variables measured appropriate to the aims of the study?	Yes	Yes	Yes	Yes
9. Were the risk factor and outcome variables measured correctly using instruments/ measurements that had been trialled, piloted or published previously?	Yes	Yes	Yes	Yes
10. Is it clear what was used to determined statistical significance and/or precision estimates? (eg, p values, CIs)	Yes	Yes	Yes	Yes
11. Were the methods (including statistical methods) sufficiently described to enable them	Yes	Yes	Yes	Yes
12. Were the basic data adequately described?	Yes	Yes	Yes	Yes
13. Does the response rate raise concerns about non-response bias?	No	ND	ND	ND
14. If appropriate, was information about non-responders described?	Yes	ND	ND	ND
15. Were the results internally consistent?	Yes	Yes	Yes	Yes
16. Were the results for the analyses described in the methods, presented?	Yes	Yes	Yes	Yes
17. Were the authors' discussions and conclusions justified by the results?	Yes	Yes	Yes	Yes
18. Were the limitations of the study discussed?	Yes	Yes	Yes	Yes
Other				
19. Were there any funding sources or conflicts of interest that may affect the authors' interpretation of the results?	No	No	No	No
20 Was ethical approval or consent of participants attained?	Yes	Yes	Yes	Yes

† Power analysis was not performed but the sample is based on the national population-based Korea National Health and Nutrition Examination Survey.

ND: Not determined.
